# Suitability of skin traction combined with braces for treating femoral shaft fractures in 3–5 years old children

**DOI:** 10.1186/s13018-023-03547-5

**Published:** 2023-01-23

**Authors:** Menglei Wang, Yuxi Su

**Affiliations:** Department of Orthopedics, Chongqing Key Laboratory of Pediatrics; Ministry of Education Key Laboratory of Child Development and Disorders; National Clinical Research Center for Child Health and Disorders; China International Science and Technology Cooperation base of Child development and Critical Disorders, Children’s Hospital of Chongqing Medical University, 136# Zhongshan 2 Road, Yuzhong District, Chongqing, 400014 China

**Keywords:** Skin traction, Braces, Femoral shaft fractures, Children, Preschool

## Abstract

**Background:**

In children aged 3–5 years, femoral fractures are common and are frequently treated using flexible intramedullary nails (FIN) or spica casting. Recently, more surgeons have been relying on FIN surgery because of the high rate of complications associated with spica casts, such as skin irritation and re-adjustment surgery. We aimed to evaluate the effect of skin traction combined with braces in 3–5 years old children at our hospital.

**Methods:**

We retrospectively analyzed 125 children aged 3–5 years with femoral shaft fractures treated at our hospital between January 2010 and December 2020. We assigned 68 patients who underwent FIN surgery to Group A and 57 patients treated with skin traction and braces to Group B. Comparative analysis included the children’s age, sex, side of the affected limb, cause of fracture, function of the knee joint, healing time of the fracture, duration of hospitalization, cost of hospitalization, and complications. The complications evaluated included joint dysfunction, pain, infection, pressure ulcers, angular deformities, limb length differences, re-fractures, nonunion fractures, and delayed union.

**Results:**

There were significant differences in and hospital costs (*p* = 0.001). Conversely, no statistically significant differences were observed in sex (*p* = 0.858), injury type (*p* = 0.804), age (*p* = 0.231), hospitalization time (*p* = 0.071), bone healing time (*p* = 0.212), and complications. Pressure ulcers, nonunion fractures, and delayed union did not occur in both groups.

**Conclusion:**

Both methods had similar therapeutic effects and postoperative complications in children aged 3–5 years with femoral shaft fractures. Therefore, skin traction combined with braces is recommended for this population and for patients hospitalized in institutions where several beds are available, with a consequent possibility of prolonged hospitalization.

*Level of Evidence*: IV.

## Introduction

Femoral shaft fracture (FSF) is one of the most common fractures in children, accounting for approximately 1.6% of pediatric fractures [1, 2]. Spica casts have been used for the conservative treatment of FSF for several decades [3, 4]. With the development of surgical techniques, particularly the flexible intramedullary nail (FIN) fixation method, most surgeons rely on surgery rather than treatment with spica casts [5]. Increasing evidence has proven that hip braces or FIN fixation are better for treating FSF in children aged 3–5 years [6–11]. However, there are also some disadvantages associated with FIN fixation. First, this procedure requires surgery and induction of anesthesia twice, initially for the fixation and subsequently for the FIN material removal. Second, the cost of hospitalization for FIN fixation is much higher than that associated with spica casting. Third, scars are formed if the surgeon fails to achieve close reduction. Fourth, other surgery-related complications, such as infection of the incision and pin end irritations, may occur. Therefore, the best treatment for FSF in children aged 3–5 years remains controversial.

Compared with spica casting, hip bracing has the advantages of being lightweight, with good air permeability and ease of care [12]. Hip brace was much more comfortable for patients than spica casting, as the humid weather was much severe in South of our country. Second, patients with skin traction in hospital can be observed closely by clinical physical examination, X-radiographs, ultrasound. Once the patient had fracture displacement, immediate adjustment can be performed for the patients. The patients’ guardians will be satisfied with procedure. Third, skin traction had no incision or without sedation or anesthesia. However, braces have also been associated with more fracture displacements [13]. In this study, we combined skin traction and brace fixation during hospitalization. To our knowledge, this is the first comparative study of skin traction combined with braces.

## Patients

We retrospectively enrolled 125 patients aged 3–5 years with FSF treated at our hospital between January 2010 and December 2018. Patients (*n* = 68) who underwent FIN fixation surgery were classified into Group A, while those (*n* = 57) who underwent skin traction combined with braces were classified into Group B. General data were retrieved from the hospital database, and clinical results were collected during follow-up. Comparative analysis included the children’s age, sex, side of the affected limb, cause of fracture, knee joint function, fracture healing time, complications, length, and cost of hospitalization. The complications evaluated included joint dysfunction, pain, infection, pressure ulcers, angular deformities, limb length differences, re-fractures, nonunion fractures, and delayed union.

The inclusion criteria were as follows: (1) age 3–5 years with freshly closed FSF (< 2 weeks following injury), (2) confirmed diagnosis by radiography or computed tomography, (3) fixation with elastic intramedullary nail or brace treatment, and (4) follow-up period ≥ 12 months. In contrast, the exclusion criteria were as follows: (1) fractures of other parts or dislocations of the hip and knee joints, (2) nerve or vascular injury, (3) infection or received antibiotic treatment at the time of admission, (4) pathological fractures, and (5) important preexisting ipsilateral limb disease or comorbidity.

## Methods

### Group A

The surgical method for Group A was as follows: (1) the patient was placed in a supine position, general anesthesia was administered along with intravenous and tracheal intubation, and the affected limb underwent abduction and traction; (2) a longitudinal incision (~ 2 cm) was made in the distal metaphysis of the femur, and an FIN was driven to the fractured end; (3) closed reduction was performed first; (4) the elastic intramedullary nail was pushed through the fracture line to the proximal end of the fracture; and (5) using the same technique, another nail was pushed from the distal femoral metaphysis away from the epiphyseal plate through the fracture line to the proximal end of the fracture. If the reduction was unsuccessful, a small incision (~ 5 cm) was made in the skin above the fracture. Subsequently, the fracture end was reduced under direct vision; (6) reduction and fixation of the fracture was verified using an X-ray. The nail protruded by approximately 0.5–1.0 cm at the distal end, which was a convenient placement for later removal.

The ethics committee at our hospital approved this study, and we obtained written informed consent from all the parents/guardians of the children involved in the study. The study was conducted in accordance with the Declaration of Helsinki. The same team of orthopedic surgeons at our hospital completed all treatments in this study.

#### Postoperative treatment

**Fig. 1:**
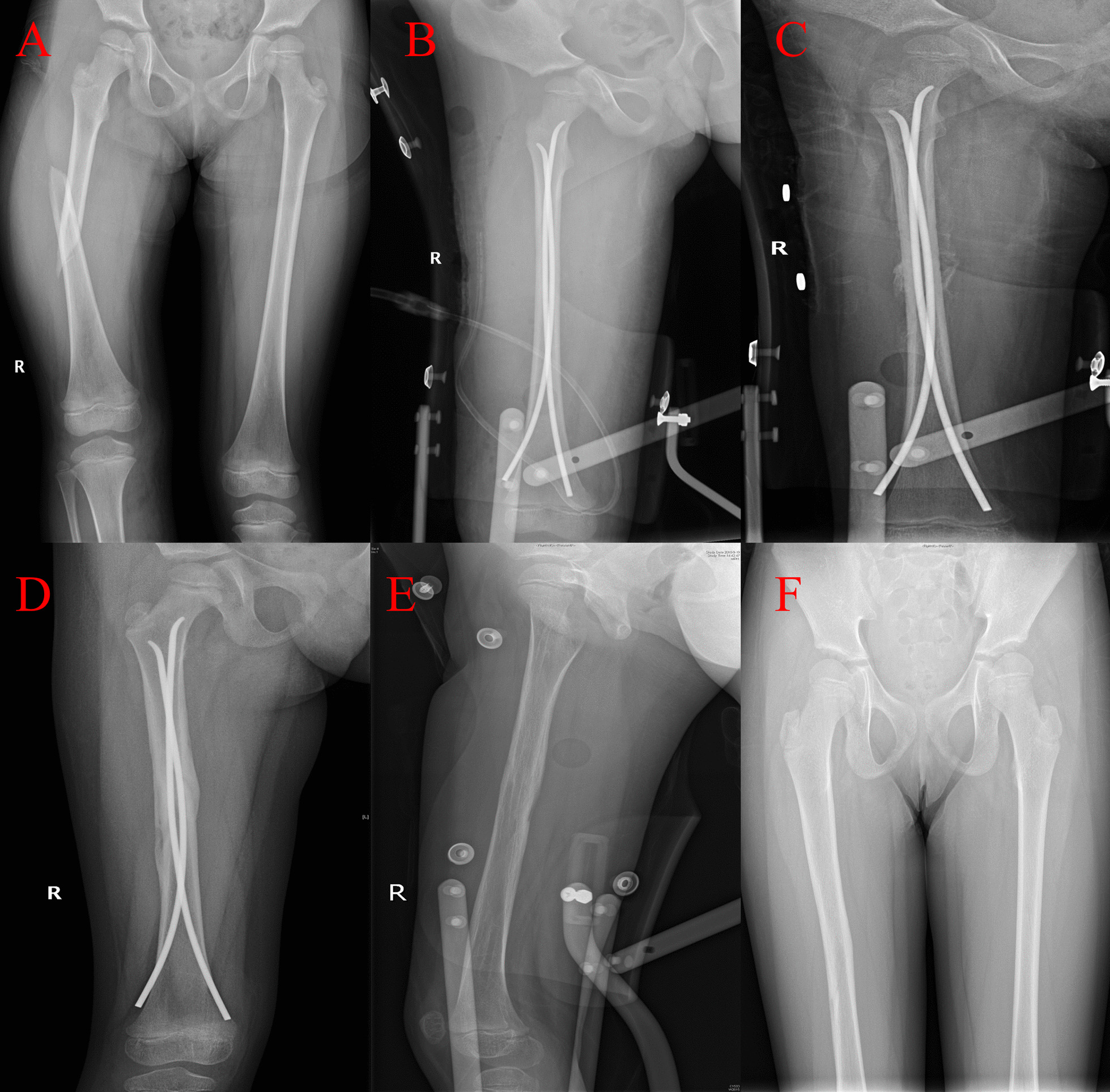
A 5-year-old male injured in a car accident and treated using FIN. **A** AP radiographs of the right femoral fracture caused by the accident. **B** Postoperative AP radiograph showing FIN fixation. **C** AP radiograph at 1 month postoperatively. **D** AP radiograph at 3 months postoperatively. **E** AP radiograph at 7 months postoperatively. **F** AP radiograph at 18 months postoperatively. *AP* Anteroposterior; *FIN* Flexible intramedullary nails

In all cases, a radiograph of the affected femur was reviewed weekly during the first month following surgery. After 2–3 weeks, the children and their parents were instructed to properly move the adjacent joints and begin partial weight-bearing for approximately 4 weeks. When a bridging callus appeared and the fracture line was no longer visible on the X-ray film, partial weight-bearing progressed to full weight-bearing. The time for initiation of weight-bearing exercises was determined based on the fracture type and the X-ray results during the follow-up period. The alignment and healing of the fractures were reviewed using radiographs at 1, 3, and 6 months postoperatively. The children were evaluated clinically and radiologically at each follow-up examination, and the complications were recorded. When the fracture was fully healed (2.9 ± 0.9 months), the internal fixation device was removed under general anesthesia in the operating room (Fig. 1).

### Group B

**Fig. 2:**
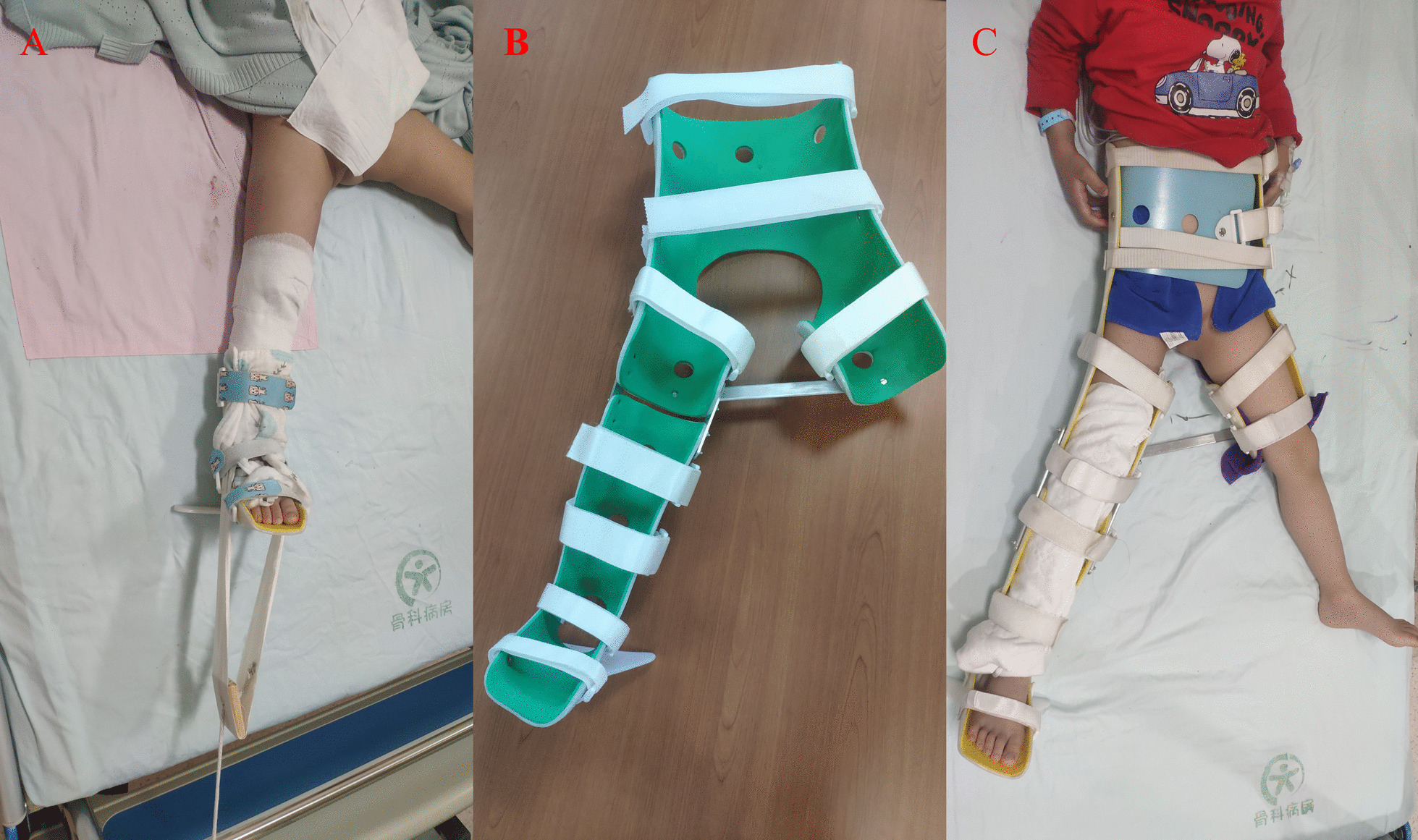
Skin traction and custom-made spica brace. **A** Skin traction of a 5-year-old male patient. **B** Appearance of the custom-made spica brace. **C** The custom-made spica brace was used to fix the patient

**Fig. 3:**
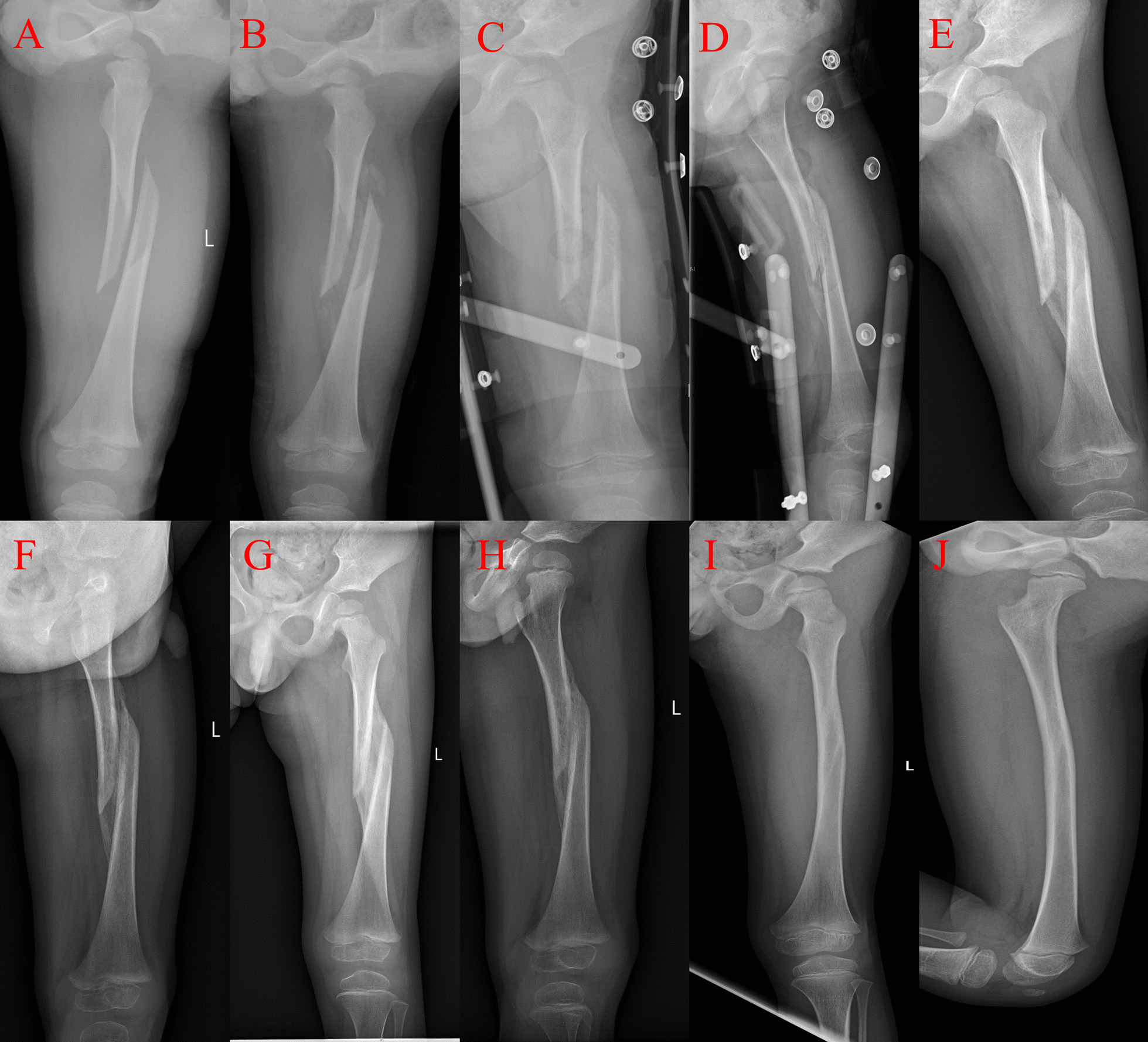
A 3-year-old male injured after a fall was treated using skin traction combined with a spica brace. **A** AP radiograph of the left femoral fracture by skin traction on day 8. **B** Bone callus as observed on AP radiograph by skin traction and then, spica brace on day 15. **C**, **D** Bone callus progressed further on AP and lateral radiographs. The spica brace is still visible on day 24. **E**, **F** AP and lateral radiographs show femur fracture remodeling at 3 months postoperatively. **G**, **H** AP and lateral radiographs show femur fracture remodeling at 4 months postoperatively. **I**, **J** AP and lateral radiographs show femur fracture remodeling at 15 months postoperatively

Patients in Group B were first enrolled at the hospital, and skin traction was performed at their bedside (Fig. 2A). The fractures were observed using radiographs and adjusted according to the displacement of the fractures. The fractures were examined every 3–5 days until a bone callus appeared on the radiographs. Subsequently, the custom-made spica braces were applied (Fig. 2B, C), and the patients were discharged from the hospital. Follow-up examinations were conducted every 1 or 2 weeks with the patients as outpatients. If the radiographs revealed that the callus was strong enough, the fracture gradually became stable, and there were more calluses at the fracture end. Subsequently, the brace was removed, and functional exercise was initiated. Normal walking and activities were gradually restored based on the healing of the fracture. The follow-up procedure for Group B was the same as that for Group A (Fig. 3).

### Outcome evaluation

Based on the Knee Society Score method in evaluating treatment effects among children [7], the ratings were classified as follows: (1) excellent: the knee joint was fully extended, 120° flexion, no pain, and shortening < 1 cm; (2) good: fully extended, 90° flexion, no or occasionally mild pain, slight angulation, and shortening was < 2 cm; (3) medium: extension difference of 10°, range of motion > 60°, frequent mild pain, internal and external angle < 10°, shortening < 3 cm; or (4) poor: extension difference of 10°, range of motion < 60°, pain was obvious and long-lasting, the inner and outer angles were > 10°, and shortening was > 3 cm.

### Statistical analysis

Statistical analyses were conducted using SPSS 20.0 (IBM Corp., Armonk, NY, USA), and values are expressed as means ± standard deviations or numbers and percentages. Continuous variables were compared using the *t* test, and the χ^2^ test was used for the categorical variables. Statistical significance was set at *p* < 0.05.

## Results

Demographic characteristics of patients are listed in Table1. Fractures caused by falls and traffic accidents accounted for 50.0% (34/68) and 33.8% (23/68) of the patient, respectively, in Group A. In Group B, 63.2% (36/57) resulted from falls, and traffic accidents caused 28.1% (16/57). There were no significant between-group differences regarding AO classification (*p* = 0.804), sex (*p* = 0.858), side of the affected limb (*p* = 0.922), fracture causes (*p* = 0.270), (Table 1). The average age of children was not statistically significant (*p* = 0.231) (Table 1).

**Table 1:** Demographic characteristics of patients

	Group A (FIN)	Group B (Skin traction + Spica braces)	*p*-value
*Age*			
Mean age (years)	4.1 ± 0.9	3.5 ± 0.7	0.231
Patients quantity (*N*)	68	57	
*Sex*			
Male	44 (64.7%)	36 (63.2%)	0.858
Female	24 (35.3%)	21 (36.8%)	
*Injured femur*			
Left side	34 (50.0%)	29 (50.9%)	0.922
Right side	34 (50.0%)	28 (49.1%)	
*Injury mechanism*			
Fall	34 (50.0%)	36 (63.2%)	0.270
Pedestrian injury by vehicles	23 (33.8%)	16 (28.1%)	
Others	11 (16.2%)	5 (8.8%)	
*Fracture type (AO classification)*			
A1	19 (27.9%)	16 (28.1%)	0.804
A2	32 (47.1%)	24 (42.1%)	
A3	17 (25.0%)	17 (29.8%)	
Hospitalization cost (US dollar)	3647.0 ± 1940.7	1771.9 ± 667.1	0.001#
Duration of hospitalization (days)	16.3 ± 4.9	22.5 ± 6.3	0.071
Bone healing time (months)	2.9 ± 0.9	2.4 ± 0.9	0.212
Follow-up time (years)	4.5 ± 3.7	3.8 ± 2.7	0.556
*Knee function (Knee Society Score)*			
Excellent	59 (86.8%)	54 (94.7%)	0.151
Good	5 (7.4%)	3 (5.3%)	
Fine	4 (5.9%)	0 (0.0%)	
Poor	0 (0.0%)	0 (0.0%)	
*Complications*			
Knee function limitation	2	2	0.621
Pain	5	2	0.299
Bone deformity	3	3	0.573
Limb length discrepancy	4	2	0.427
Infections	3	0	0.108
Re-fracture	1	0	0.358
Nonunion or delayed union	0	0	

*FIN* Flexible intramedullary nails

^#^*p* < 0.05 was considered significant

There was a statistically significant difference in the average hospitalization cost (*p* = 0.001) between the two groups. There was no statistically significant difference in the good knee joint’s function rate (*p* = 0.151), hospitalization time (*p* = 0.071), and fracture healing time (*p* = 0.212) between the two groups (Table 1).

Three (3/68, 4.4%) children in Group A had wound infections postoperatively, which were resolved with appropriate antibiotic therapy. Two (2/68, 2.9%) children experienced joint dysfunction, which mainly manifested as joint stiffness and difficulty in flexion; normal function was restored after rehabilitation training. Five (5/68, 7.4%) children had skin irritation at the nail tail with local skin swelling that disappeared after removing the intramedullary nail. Three (3/68, 4.4%) children developed angulation deformity (internal and external angle > 10°) after the operation, and four (4/68, 5.9%) children developed ipsilateral limb lengthening (all < 1.5 cm), which barely affected limb function. One (1/68, 1.5%) child experienced re-fracture at the surgical site due to trauma. Two patients (2/57, 3.5%) experienced long-term pain due to brace inadaptability, and three (3/57, 5.3%) developed angulation deformity (internal and external angle > 10°) postoperatively. Additionally, two children (2/57, 3.5%) had elongated affected limbs (both < 1.5 cm), which barely affected their limb function. During subsequent follow-up visits, the activities of the children’s affected limbs returned to normal. There were no statistically significant differences regarding complications, such as joint dysfunction (*p* = 0.621), pain (*p* = 0.299), angular deformity (*p* = 0.573), and limb discrepancy (*p* = 0.427) between the two groups. (Table 1).

## Discussion

This study confirmed that skin traction combined with braces was an appropriate treatment choice for FSF in children aged 3–5 years. Currently, the choice of non-surgical or surgical treatment of FSF for children in this age group remains controversial. It is recommended that children with FSF aged 6 months–5 years should be treated with plaster fixation [1, 6, 14, 15]. However, skin complications and loss of reduction are more common with plaster fixation [3, 4]. At our center, we were reluctant to choose the spica cast for the following reasons: (1) the weather was very humid, and many patients had skin irritations, which felt very itchy. (2) the fracture may be displaced during follow-up. The patient’s guardians would not tolerate this situation, which could even lead to lawsuits.

Compared to spica casts, braces were comfortable with less skin irritation [16, 17]. However, an obvious disadvantage was that the fixation was not as stable as the casts. Thus, the occurrence of displacement was higher than that with the use of a cast; therefore, in this study, we did not use the spica cast during the first treatment of FSF. Our method of skin traction combined with a spica brace resulted in fewer complications. First, when the patients were hospitalized for skin traction, the fractures were monitored using X-rays, and they could be adjusted immediately if the displacement was unacceptable. Once the bone callus grew and the fractures were stable, the spica casts were applied.

Some studies shown that compared with spica casts, elastic intramedullary nails can successfully treat 3–5 years old children with FSF, enabling faster walking recovery with the same complication rate [11, 18, 19]. To compare the effect of both treatment methods, we statistically compared clinical data, such as the side of the affected limb, fracture causes, and fracture types in both groups. No significant differences were observed in the variables between the two study groups (Table 1). To the best of our knowledge, there have been no studies on skin traction combined with braces for the treatment of FSF in children aged 3–5 years.

Economic factors, which are particularly important in lower-income countries, were considered when treating these patients. Some studies have reported that conservative treatment was the first choice [20]. A multicenter study reported that an increasing number of surgeons performed FIN surgery in 3–5 years old children. FIN fixation costs more and requires reoperation to remove the internal fixation device [21, 22]. In this study, the cost of FIN surgery was significantly higher than that of traction combined with spica braces (*p* < 0.001). If the hospitalization time required for FIN removal was considered, no significant difference was observed (*p* = 0.122).

Some studies have reported that compared with non-surgical treatment, the use of elastic intramedullary nail fixation can enable 3–5 years old children with FSF to regain the ability to walk and return to school faster; this greatly reduces the cost of caring for the child [8, 11, 23–25]. In this study, the fracture healing times seems shorter in Group B than Group A. This was mainly because some patients underwent open reduction and fixation using FIN in Group A. Open reduction was considered a second injury for bone healing; hence, these patients could experience delayed bone callus healing. However, there was no significant difference between these groups. This may indicate that close reduction should be the first choice of treatment these patients.

Compared with hip brace fixation, elastic intramedullary nail fixation is associated with unique complications. A second operation to remove the internal fixation is necessary because of the elastic intramedullary nails [5], and skin irritation of the nail tail often causes incision pain, infection, and scars [5]. All patients in this study underwent a second operation under general anesthesia to remove the elastic intramedullary nail between 4 and 7 months postoperatively [26]. All the children's incisions healed within 2 weeks postoperatively, and no incision pain was reported. Some family members of the children were dissatisfied because of the noticeable scars resulting from the two procedures. However, cases of scar contracture affecting the children’s function were not observed.

This study showed no significant difference in postoperative knee joint function between children with elastic intramedullary nail fixation and hip brace fixation. The two groups of children experienced complications, such as joint dysfunction, pain, angular deformity, and limb length differences; however, the differences were not statistically significant. Pressure ulcers, nonunion fractures, and delayed union were not observed, and no infections or re-fractures occurred in the conservative group. Only one child with elastic intramedullary nails experienced a re-fracture at the surgical site due to trauma. Therefore, both treatment methods are acceptable. Both elastic intramedullary nail fixation and hip spica braces can fulfill the treatment requirements of 3–5 years old children with FSF.

Overall, our method of using skin traction combined with brace fixation did not require anesthesia using our method, and it was much more economical than surgery. We recommend that this method could be adopted for 3–5 years old children with FSF because it has almost similar clinical outcomes as surgery. In this study, patients aged 3–5 years, who were reluctant to undergo anesthesia and skin incision and could even afford the costs were advised to choose our method.

This study had some limitations. Generally, certain types of fractures tend to be treated conservatively or surgically. This may be influenced by the surgeons' advice or other reasons. In this study, we excluded patients who were only suitable for surgery or conservative treatment; however, both groups of patients can be treated with surgery or conservative treatment, and the final decision was made by the patients’ guardians. Some bias would still have been observed between the study groups even if the fracture types and other clinical data were compared. It is important to evaluate the societal cost; however, it was difficult to calculate it in this study because some of the caregivers were lost to follow-up. The other limitation was the lack of a control group treated with spica casts because this treatment method is no longer used at our facility. And for the compilations, the quantity of the patients was small, it cannot be compared by statistical analysis. Additionally, this was a single-center, retrospective study; therefore, prospective, multicenter studies are required in the future.

## Conclusion

Skin traction combined with spica braces is a safe and feasible method for treating femoral fractures in children aged 3–5 years. This treatment method could be an appropriate choice, particularly for lower-income countries and for patients hospitalized in institutions where several beds are available and, therefore, the hospitalization period can be prolonged.

## Data Availability

The datasets used and/or analyzed during the current study are available from the corresponding author on reasonable request.

## Abbreviations

FIN: Flexible intramedullary nails
FSF: Femoral shaft fracture
